# T-Cell Large Granular Lymphocyte Leukemia: An Interdisciplinary Issue?

**DOI:** 10.3389/fonc.2022.805449

**Published:** 2022-02-10

**Authors:** Johanna Schreiber, Alexander Pichler, Christoph Kornauth, Hannes Kaufmann, Philipp B. Staber, Georg Hopfinger

**Affiliations:** ^1^ Department of Internal Medicine III, Division of Hematology and Oncology, Klinik Favoriten, Vienna, Austria; ^2^ Department of Medicine I, Division of Hematology, Medical University of Vienna, Vienna, Austria; ^3^ Department of Pathology, Medical University of Vienna, Vienna, Austria

**Keywords:** large granular lymphocyte (LGL), T-LGL leukemia, STAT3, rheumatoid arthritis, neutropenia, splenomegaly

## Introduction

Large granular lymphocytic leukemia (LGLL) is an indolent and rare lymphoproliferative disorder of mature cytotoxic T-cells or Natural Killer (NK)-cells accounting for 2-5% of chronic lymphoproliferative disorders in North America and Europe (1, 2).

LGLL is associated in up to 15-40% with autoimmune disorders, with rheumatoid arthritis (RA) being the most common (10-18%). Rheumatoid factor (RF) and antinuclear antibody (ANA) are detected in about half of the patients (1). As symptoms are nonspecific, diagnosis can be delayed. A close collaboration with a specialist in hematology is recommended.

According to the WHO classification 2017 (3), LGLL is divided into T-LGL leukemia (T-LGLL, 85%), chronic lymphoproliferative disorder of NK-cells (CLPD-NK, 10%) and the more aggressive NK-LGL leukemia (ANKL, 5%). T-LGLL and CLPD-NK have a median age of 60 years and tend to have an indolent course, whereas aggressive NK-LGL leukemia more often affects younger patients and is highly associated with EBV (3–6).

LGL leukemia (LGLL) should be considered in patients with marked neutropenia, lymphocytosis, recurrent infections, anemia and autoimmune disorders. Typical “B” symptoms are seen in only 20-30% of LGLL patients (7). Most patients with T-LGLL present with chronic neutropenia resulting in recurrent infections but courses without any infections are possible (1, 8, 9). Lymphocytosis is observed in about 50%, thrombocytopenia in < 25% and anemia in 10-30% of LGL patients. Splenomegaly is seen in about a quarter of patients, whereas hepatomegaly and lymphadenopathy are rare (1, 2, 8, 10).

**Figure 1:**
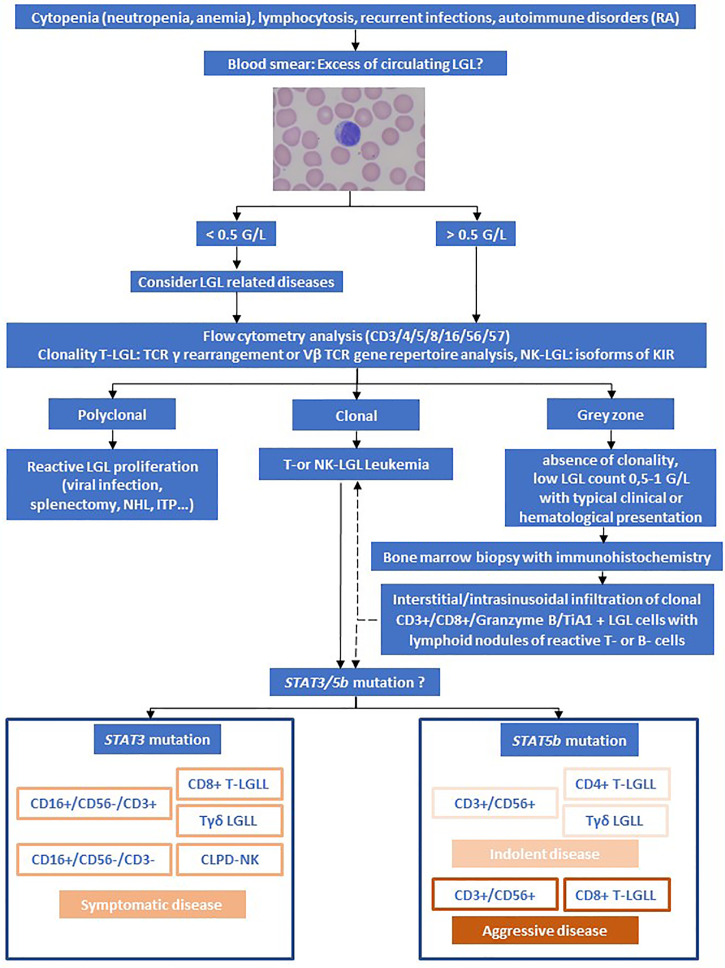
Algorithm for the evaluation of LGL Leukemia. Adapted from Lamy et al. and Teramo et al. (1, 11). Rheumatoid arthritis (RA), Non-Hodgkin Lymphoma (NHL), immune thrombocytopenia (ITP).

Diagnosis is based on cytology (blood smear), flow cytometry of peripheral blood and detection of clonality of T-cell receptor (TCR) rearrangement (see **Figure 1**).

Large granular lymphocytes represent a morphological subtype that are larger (15-18µm) than most circulating lymphocytes (7-10µm). LGL cells show an abundant cytoplasm containing prominent azurophilic granules and a round or reniform nucleus with mature chromatin (see **Figure 1**) (9).

Most patients present with a persistent increased number of circulating LGL ranging from 1-6 G/L. According to the 2017 WHO classification (12), a threshold of > 2 G/L (normal: <0.3 G/L) persistent circulating LGLs for more than 6 months is mandatory. However, numerous patients have a lower number of clonal LGLs, typically presenting with other clinical or hematologic features such as RA or cytopenia. Accordingly, cases with LGL counts of <2 G/L meeting all other criteria are consistent with diagnosis as well (13).

The majority of T-LGL cells are CD3+, CD8+, CD16+, CD57+, CD45RA+, TCRαβ+, and CD4-, CD56-, CD27-, CD45R0-, CD28-, CD62L-, CD5^dim^ and/or CD7^dim^. Rarely LGLL is CD4+ with or without coexpression of CD8. NK-LGL leukemia and NK-LGL lymphocytosis are characterized by the following phenotype: CD2+, CD3−, CD3ϵ+, TCRαβ−,CD4−, CD8+, CD16^+^, CD56+, CD57^+/-^ (1).

Diagnosis is confirmed by detection of TCR rearrangement by PCR allowing distinguishing reactive LGL proliferation from real leukemic proliferation. The majority are αβ variants, while 10% are γδ variants (14). Clonality can also be assessed by flow cytometry for different TCR chain domains (Vβ, Vγ, Vδ) using various antibodies. The current Vβ mAbs panel covers 65% of the Vβ spectrum (15). Detection of γδTCR and its subtypes (Vδ1 and Vδ2) at protein level by flow cytometry represents a fast practical method for determining the clonality of γδ T-cells (16). As NK-LGL do not express TCR, restricted expression of activating isoforms of killer immunoglobulin-like receptor (KIR) can be used (17).

Bone marrow aspirate and/or biopsy with immunohistochemistry is not routinely recommended but can support the diagnosis in uncertain cases. Typical features observed in case of bone marrow infiltration of LGLL are hypercellularity with individual or small clusters of LGLs localized primarily in sinusoids. Often, reactive, predominantly CD20+ B-lymphoid aggregates are seen with peripherally accentuated CD3+ T-cells. Expression of cytotoxic markers TiA1, granzyme B and granzyme M are considered characteristic histopathologic findings of LGLL (18–21).

As T-LGLL can mimic other T-cell lymphoid malignancies, careful differentiation from lymphomatous and leukemic disorders affecting T-cells e.g. CLPD-NK, ANKL and from conditions with reactive LGL expansions, is required. Several conditions can lead to the development of reactive LGL proliferation, including viral infections (e.g. HIV, CMV, EBV, HBV and HCV), hemophagocytic syndrome, immune thrombocytopenia (ITP), non-Hodgkin lymphoma (NHL), solid tumors, splenectomy. These are typically poly- or oligoclonal (2, 7).

Furthermore, differentiation from Felty syndrome with typical triad of rheumatoid arthritis, neutropenia and splenomegaly might be difficult (1, 19, 20, 22, 23).

The etiology of T-LGL leukemia is still unknown. It is believed that the initial step relies on chronic antigen exposure leading to dysregulation of apoptosis, mainly due to dysregulation of the JAK/STAT pathway (1). Constitutive activation of STAT3 is often related to STAT3 gain of function mutations in 30-40% of T-LGLL (24, 25). *STAT5b* mutation is less frequent (2%) but highly prevalent in the rare subset of CD4+ T-LGL (1, 26–29). Therefore, mutations in *STAT3* and *STAT5b* were included in the 2017 WHO classification of LGL disease (3, 12). In addition, proinflammatory cytokines such as platelet-derived growth factor and IL-6, IL-12, IL-15 contribute to leukemic LGL persistence and proliferation (30). Interestingly, Felty syndrome might be associated with somatic *STAT3* mutations indicating a potential common pathogenesis (23). *STAT3* and *STAT5b* mutation might have an impact on clinical outcome, as *STAT3* mutation is associated with symptomatic disease and a specific phenotype: CD16+, CD56-, CD8+, Tγδ. Additionally, the immunophenotypic signature CD56^neg/dim^/CD16^+^/CD57^-^ in CLPD-NK patients is associated with a more symptomatic disease and the presence of *STAT3* mutation (31). T-LGLL harboring a *STAT5b* mutation and being CD3+, CD8+, CD56+, CD16– and CD57– shows a more aggressive course with poor prognosis, whereas expression of CD4 and CD56 antigens as well as CD56, CD3, Tγδ-LGLL are often associated with a more indolent course (11, 27).

To illustrate our proposed algorithm (see **Figure 1**), we will further discuss two clinical cases of LGL-Leukemia.

## Case Reports

### Indolent Course of a γδ T-LGL-Leukemia

A 42 year-old-male was seen by a rheumatologist for joint pain. However, no rheumatologic disease was found. Due to a leukocytosis of 17.7 G/l (3.9-10.2 G/L), the patient was referred to our clinic. B-symptoms or recurrent infections were denied. Past medical history included diabetes type 2, hypertension and obesity. The physical examination was unremarkable and the ultrasound showed neither lymphadenopathy nor hepatosplenomegaly. Laboratory findings revealed an increase of absolute lymphocytes (7.1 G/L) without neutropenia, anemia or thrombocytopenia. Serologic examination showed no viral infection or autoimmune disorder (RF, ANA negative). Peripheral blood smears demonstrated an increase of predominantly mature lymphocytes occasionally with cytoplasmic azurophilic granules. Flow cytometry revealed an increase in γδ T-cells with a CD2+, CD3+, CD16+, CD56+, CD5+, CD7+ and CD4-/CD8- phenotype, which constituted approximately 45% (2.1 G/L) of T-cells. Cytogenetic study showed a normal male karyotype and a T-cell receptor γδ gene rearrangement. In the bone marrow biopsy, a diffuse interstitial and intrasinusoidal infiltration of atypical CD3+, CD5+ T-lymphocytes with expression of cytotoxic molecules TiA1 and Granzyme B was observed. *STAT3* mutation was not detected. An asymptomatic course of T-LGLL was diagnosed, prompting a watch and wait strategy with laboratory and clinical controls every 3-6 months. After three years, the patient is in continuous observation without any symptoms.

### γδ T-LGL-Leukemia Presenting With Immune Thrombocytopenia and Pure Red Cell Aplasia

A 70 year old patient presented with severe normochromic normocytic anemia with hemoglobin of 2.6 g/dL (13.5-17.2 g/dL), thrombocytopenia of 50 G/L (150-370 G/L) and normal total leukocyte and lymphocyte count. Past medical history encompassed stage II gastric carcinoma 12 years ago that was treated with gastrectomy and splenectomy, as well as perioperative chemotherapy. Thirteen months earlier to this presentation he had been admitted to the gastroenterology department due to microcytic hypochromic anemia (hemoglobin 7.5 g/dL) and thrombocytopenia (36 G/L). Bleeding as well as local recurrence were excluded by gastro-, colon- and capsule- endoscopy. Additionally, lab results showed a chronic kidney disease (CKD) with creatinine 2.31 (0.67-1.17 g/dl) and GFR 27.7 ml/min (>90 ml/min) with a concomitant iron deficiency assuming a renal anemia with substrate deficiency. The patient had received iron supplementation plus s.c. erythropoietin and had been discharged to outpatient care.

Endoscopies showed no evidence of bleeding. Next, the patient was referred to our hematology department. Neither “B” symptoms nor recurrent infections were reported. Serology revealed antibodies against glycoprotein IIb/IIa, Ib/IX confirming chronic ITP and cortisone therapy was initialized. Peripheral blood smear examination identified a slightly increased number of circulating LGL (0,985 G/L). Flow cytometry revealed an abnormal population of γδ T-cells with CD3+, CD16-, CD57^mid^, CD56^dim^, CD8^dim^ and representing 42% of T lymphocytes. A bone marrow biopsy demonstrated selective pure red cell aplasia (PRCA), signs of dysmegakaryopoiesis, and a discrete proliferation of partially intrasinusoidal localized CD8+ CD3+ and TiA1+ T-cells. Granulocytopoiesis was largely regular. Cytogenetic and fluorescence *in situ* hybridization evaluation showed a normal karyotype (46, XY) and no chromosomal or genetic aberrations ruling out other hematological malignancies e.g. myelodysplastic syndrome. No viral (Parvovirus B19, HBV, HCV, EBV, CMV) or serological (ANA, ANCA, RF) positivity were found at the initial laboratory workup. Chest and abdominal computed tomography ruled out the presence of thymoma and other malignancies. Although *STAT3* mutation was not detected, TCR gene rearrangement showed a clonal pattern of the TCRγδ. These findings were consistent with the diagnosis of T LGL-associated PRCA. Immunosuppressive therapy was indicated because of autoimmune mediated thrombocytopenia and blood cell (RBC) transfusion dependency (every 1-2 weeks). Due to the patient’s CKD, Cyclophosphamide (CP) p.o. with a dose of 50mg daily was started with careful monitoring of complete blood count to avoid myelotoxicity and prednisone therapy was continued. Erythropoietin injections were stopped. In addition, the patient received intravenous iron chelation therapy due to high ferritin levels (> 3800 μg/l). Platelet count and transfusion dependency improved and the patient is still on CP treatment. Treatment duration is planned for 6-12 months.

### Treatment Considerations and Discussion

As most patients with T-LGLL have an indolent course, only half of patients require systemic treatment at the time of diagnosis and overall survival at 10 years is 70%. In asymptomatic patients, a watch and wait strategy with laboratory and clinical controls every 6 months is suggested. Treatment is only indicated in case of symptomatic disease or impaired blood values as follows: Severe neutropenia ANC <0.5 G/L or neutropenia-associated infections, anemia hemoglobin <10 g/dL or need for RBC transfusion, thrombocytopenia with platelets <50 G/L, symptomatic autoimmune diseases, symptomatic splenomegaly, and severe B-symptoms. The main goal of treatment is relief of symptoms, reduction of infections and transfusion independence. Disease related deaths are primarily related to severe infections occurring in <10% of patients. However LGLL is not curable by conventional treatment (1, 22, 32).

Immunosuppressive therapy such as methotrexate (MTX), cyclophosphamide (CP), and cyclosporine (CsA) either alone or in combination with prednisone remains the backbone of the treatment for LGL leukemia (1, 22). Initial response might be quicker when adding prednisone but has no impact on eradication of LGL clones (4). As therapy responses might be delayed, patients should be treated for at least 4 months before response assessment (1, 22). Whether MTX or CP should be given as first line therapy is not clear. To clarify this situation, a phase II randomized trial comparing first-line MTX versus CP (NCT01976182) is ongoing (26).

MTX is often preferred in the setting of neutropenia and/or rheumatoid arthritis. It is used p.o. or i.v. weekly in a dose of 10 mg/m (2) and can be continued indefinitely if tolerated. Response is achieved in approximately 55% with time to response ranging from 2 to 12 weeks and a median duration of response ranging from 2 to 4 years. In case of severe neutropenia, oral prednisone (1 mg/kg per day) is administered in addition to MTX for the first month and tapered off by the end of the second month (22).

For CP, in a dose of 50-100 mg/m (2), response rates 55–66% are described. Treatment is limited to no more than 12 months (33). Case series demonstrated response rates to CP ranging from 60-100% in LGLL-associated PRCA (34).

If primary therapy is ineffective, a switch between MTX and CP is suggested (1). Analysis of a French cohort with 229 patients of LGL showed in 11/15 cases a clinical response with CP failed treatment of MTX (4). CsA is mostly reserved for the treatment of resistant disease (24). Dose ranges from 2–10 mg/kg/day, mostly 3 mg/kg/day and it shows an ORR of 56% and maintained as long as is it reasonably tolerated (4, 22, 24).

Other second-line agents are bendamustine, purine analogs and alemtuzumab (25, 32). Alemtuzumab, an anti-CD52 monoclonal antibody, demonstrated an ORR of 74% in a small phase II trial. However, due to toxicity, its use is limited to refractory cases and prophylactic antibiotics and CMV monitoring are necessary (1, 22, 35). Purine analogs (e.g. 2-chlorodeoxyadenosine, pentostatin and fludarabine) display a high ORR of 80% with a short period of treatment (1-3 courses) and the potential of inducing durable remission. However, data is limited and based on small case series and case reports (22, 33, 36–39).

There is no consensus regarding clinical management of aggressive forms of LGLL. Clinical behavior is close to aggressive leukemia and some clinicians propose a CHOP-like based or cytosine arabinoside-containing polychemotherapy, followed by autologous or allogeneic hematopoietic cell transplantation (1, 32, 40).

Considering the pathogenesis of LGL leukemia, various specific inhibitors were evaluated in T-LGLL. Tofacitinib, a JAK3-specific inhibitor, showed in T-LGLL patients an improvement of RA symptoms and a hematological response in 6/9 (67%) cases (26, 41). BNZ-1 a multi-cytokine inhibitor that inhibits interleukin (IL)-2, IL-15 and IL-9 signaling showed promising results in reducing cytokine mediated cell survival being investigated in a phase I/II trial (42). However, results are pending. The histone deacetylase (HDAC) inhibitor Belinostat has recently demonstrated a marked activity in refractory T-LGL (43). Interestingly, anti-CD20 MoAb Rituximab showed promising response in RA-associated LGL-leukemia (44).

Our first patient had a rare subtype of T-LGL with a specific phenotype: CD3+, CD16+, CD56+ and CD5+ but CD4-/CD8-. Regarding differential diagnoses, CD4-/CD8- T-LGL displays an immunophenotype and clinical pattern overlapping with the aggressive lymphoma hepatosplenic T-cell lymphoma (HSTCL). (45) ^(p4)^As HSTCL is usually CD5 and CD57 negative, it is helpful in distinguishing it from Tγδ-LGLL (46). Moreover, in contrast to described cases in literature, our patient showed an asymptomatic course without splenomegaly or autoimmune cytopenia (11, 27, 47). A *STAT3* mutation was not detected. According to Teramo et al., CD3+, CD56+ and Tγδ- LGLL seems to correlate with an indolent presentation, which is compatible with the immunophenotypic profile and indolent course of our patient (11).

Our second patient with a γδ-T cell subpopulation being CD3+, CD16-, CD57^mid^, CD56^dim^ γδ-T cells showed a symptomatic course with ITP and PRCA. T-LGL is seen in 15% to 20% of patients with PRCA (48). Frequent red blood cell transfusions caused iron overload. Treatment with cortisone and CP resulted in transfusion independence and further confirmed the therapeutic potential of CP for T-LGLL combined with PRCA. The precise underlying mechanism of CP in LGLL-associated PRCA is still not known. It is suspected to work by reducing cytotoxic T-lymphocytes that damage antibody-bound erythroblasts directly (49).

In conclusion, LGL is a rare disease and prospective data are scarce. Diagnosis of LGL is complex and oligosymptomatic clinical presentation can delay diagnosis. Patients with LGL cells as described above should prompt a careful workup to rule out reactive LGL expansion from clonal LGL leukemia. Differential blood count, blood smear, immunophenotyping and TCR-rearrangement analysis are mandatory. If diagnosis of LGLL is confirmed, close controls depending on severity of either symptoms or lab findings are necessary for patients requiring therapy. However, the majority of cases are indolent and close monitoring is necessary.

## Author Contributions

JS wrote the manuscript. JS, AP, CK, PS, HK, and GH contributed to the manuscript preparation and have read and approved all drafts. GH reviewed and approved the final version of the manuscript. All authors contributed to the article and approved the submitted version.

## Conflict of Interest

The authors declare that the research was conducted in the absence of any commercial or financial relationships that could be construed as a potential conflict of interest.

## Publisher’s Note

All claims expressed in this article are solely those of the authors and do not necessarily represent those of their affiliated organizations, or those of the publisher, the editors and the reviewers. Any product that may be evaluated in this article, or claim that may be made by its manufacturer, is not guaranteed or endorsed by the publisher.

## References

[1] Lamy T Moignet A Loughran TP. LGL Leukemia: From Pathogenesis to Treatment. Blood (2017) 129(9):1082–94. doi: 10.1182/blood-2016-08-692590 28115367

[2] Loughran TP. Clonal Diseases of Large Granular Lymphocytes. Blood (1993) 82(1):1–14. doi: 10.1182/blood.V82.1.1.bloodjournal8211 8324214

[3] Swerdlow S Campo E Harris NL Jaffe ES Pileri SA Stein H. WHO Classification of Tumours of Haematopoietic and Lymphoid Tissues (2017). Available at: https://publications.iarc.fr/Book-And-Report-Series/Who-Classification-Of-Tumours/WHO-Classification-Of-Tumours-Of-Haematopoietic-And-Lymphoid-Tissues-2017.

[4] Bareau B Rey J Hamidou M Donadieu J Morcet J Reman O. Analysis of a French Cohort of Patients With Large Granular Lymphocyte Leukemia: A Report on 229 Cases. Haematologica (2010) 95(9):1534–41. doi: 10.3324/haematol.2009.018481 PMC2930955 20378561

[5] Poullot E Zambello R Leblanc F Bareau B De March E Roussel M. Chronic Natural Killer Lymphoproliferative Disorders: Characteristics of an International Cohort of 70 Patients. Ann Oncol Off J Eur Soc Med Oncol (2014) 25(10):2030–5. doi: 10.1093/annonc/mdu369 PMC4176455 25096606

[6] Lim MS de Leval L Quintanilla-Martinez L. Commentary on the 2008 WHO Classification of Mature T- and NK-Cell Neoplasms. J Hematop (2009) 2(2):65–73. doi: 10.1007/s12308-009-0034-z 19669191 PMC2725280

[7] Lamy T Loughran TP. Large Granular Lymphocyte Leukemia. Cancer Control J Moffitt Cancer Cent (1998) 5(1):25–33. doi: 10.1177/107327489800500103 10761014

[8] Loughran TP Kadin ME Starkebaum G Abkowitz JL Clark EA Disteche C. Leukemia of Large Granular Lymphocytes: Association With Clonal Chromosomal Abnormalities and Autoimmune Neutropenia, Thrombocytopenia, and Hemolytic Anemia. Ann Intern Med (1985) 102(2):169–75. doi: 10.7326/0003-4819-102-2-169 3966754

[9] Shah A Diehl LF St Clair EW. T Cell Large Granular Lymphocyte Leukemia Associated With Rheumatoid Arthritis and Neutropenia. Clin Immunol Orlando Fla (2009) 132(2):145–52. doi: 10.1016/j.clim.2009.03.515 19394280

[10] Hara T Mizuno Y Nagata M Okabe Y Taniguchi S Harada M. Human Gamma Delta T-Cell Receptor-Positive Cell-Mediated Inhibition of Erythropoiesis *In Vitro* in a Patient With Type I Autoimmune Polyglandular Syndrome and Pure Red Blood Cell Aplasia. Blood (1990) 75(4):941–50. doi: 10.1182/blood.V75.4.941.941 2105751

[11] Teramo A Barilà G Calabretto G Vicenzetto C Gasparini VR Semenzato G. Insights Into Genetic Landscape of Large Granular Lymphocyte Leukemia. Front Oncol (2020) 10:152. doi: 10.3389/fonc.2020.00152 32133291 PMC7040228

[12] Matutes E. The 2017 WHO Update on Mature T- and Natural Killer (NK) Cell Neoplasms. Int J Lab Hematol (2018) 40(Suppl 1):97–103. doi: 10.1111/ijlh.12817 29741263

[13] Swerdlow SH Campo E Pileri SA Harris NL Stein H Siebert R. The 2016 Revision of the World Health Organization Classification of Lymphoid Neoplasms. Blood (2016) 127(20):2375–90. doi: 10.1182/blood-2016-01-643569 PMC4874220 26980727

[14] O’Keefe CL Plasilova M Wlodarski M Risitano AM Rodriguez AR Howe E. Molecular Analysis of TCR Clonotypes in LGL: A Clonal Model for Polyclonal Responses. J Immunol Baltim Md 1950 (2004) 172(3):1960–9. doi: 10.4049/jimmunol.172.3.1960 14734782

[15] Giudice V D’Addona M Montuori N Selleri C. The Value of Flow Cytometry Clonality in Large Granular Lymphocyte Leukemia. Cancers (2021) 13(18):4513. doi: 10.3390/cancers13184513 34572739 PMC8468916

[16] Chen X Zhao S Liu L Qiao C Wang Y Fan L. Flow Cytometric Pattern of Tcrvδ Subtype Expression Rapidly Identifies γδt Cell Lymphoma. Front Oncol (2020) 10:844. doi: 10.3389/fonc.2020.00844 32612945 PMC7308429

[17] Zambello R Falco M Della Chiesa M Trentin L Carollo D Castriconi R. Expression and Function of KIR and Natural Cytotoxicity Receptors in NK-Type Lymphoproliferative Diseases of Granular Lymphocytes. Blood (2003) 102(5):1797–805. doi: 10.1182/blood-2002-12-3898 12750175

[18] Morice WG Jevremovic D Hanson CA. The Expression of the Novel Cytotoxic Protein Granzyme M by Large Granular Lymphocytic Leukaemias of Both T-Cell and NK-Cell Lineage: An Unexpected Finding With Implications Regarding the Pathobiology of These Disorders. Br J Haematol (2007) 137(3):237–9. doi: 10.1111/j.1365-2141.2007.06564.x 17408463

[19] Burks EJ Loughran TP. Pathogenesis of Neutropenia in Large Granular Lymphocyte Leukemia and Felty Syndrome. Blood Rev (2006) 20(5):245–66. doi: 10.1016/j.blre.2006.01.003 16530306

[20] Lamy T Loughran TP. Clinical Features of Large Granular Lymphocyte Leukemia. Semin Hematol (2003) 40(3):185–95. doi: 10.1016/s0037-1963(03)00133-1 12876667

[21] Osuji N Beiske K Randen U Matutes E Tjonnfjord G Catovsky D. Characteristic Appearances of the Bone Marrow in T-Cell Large Granular Lymphocyte Leukaemia. Histopathology (2007) 50(5):547–54. doi: 10.1111/j.1365-2559.2007.02656.x 17394489

[22] Lamy T Loughran TP. How I Treat LGL Leukemia. Blood (2011) 117(10):2764–74. doi: 10.1182/blood-2010-07-296962 PMC3062292 21190991

[23] Savola P Brück O Olson T Kelkka T Kauppi MJ Kovanen PE. Somatic STAT3 Mutations in Felty Syndrome: An Implication for a Common Pathogenesis With Large Granular Lymphocyte Leukemia. Haematologica (2018) 103(2):304–12. doi: 10.3324/haematol.2017.175729 PMC5792275 29217783

[24] Battiwalla M Melenhorst J Saunthararajah Y Nakamura R Molldrem J Young NS. HLA-DR4 Predicts Haematological Response to Cyclosporine in T-Large Granular Lymphocyte Lymphoproliferative Disorders. Br J Haematol (2003) 123(3):449–53. doi: 10.1046/j.1365-2141.2003.04613.x 14617004

[25] Rosamilio R Giudice V Ferrara I Annunziata S Pezzullo L Villani G. Prolonged Complete Hematologic Response in Relapsed/Refractory T-Large Granular Lymphocyte Leukemia After Bendamustine Treatment. Transl Med UniSa (2016) 15:80–3. PMC5120754 27896231

[26] Wahnschaffe L Herling M. Hijacking the Pathway: Perspectives in the Treatment of Mature T-Cell Leukemias. HemaSphere (2021) 5(6):e573. doi: 10.1097/HS9.0000000000000573 34095757 PMC8171373

[27] Andersson EI Tanahashi T Sekiguchi N Gasparini VR Bortoluzzi S Kawakami T. High Incidence of Activating STAT5B Mutations in CD4-Positive T-Cell Large Granular Lymphocyte Leukemia. Blood (2016) 128(20):2465–8. doi: 10.1182/blood-2016-06-724856 PMC5114490 27697773

[28] Barilà G Teramo A Calabretto G Vicenzetto C Gasparini VR Pavan L. Stat3 Mutations Impact on Overall Survival in Large Granular Lymphocyte Leukemia: A Single-Center Experience of 205 Patients. Leukemia (2020) 34(4):1116–24. doi: 10.1038/s41375-019-0644-0 31740810

[29] Kawakami T Sekiguchi N Kobayashi J Imi T Matsuda K Yamane T. Frequent STAT3 Mutations in CD8+ T Cells From Patients With Pure Red Cell Aplasia. Blood Adv (2018) 2(20):2704–12. doi: 10.1182/bloodadvances.2018022723 PMC6199660 30337298

[30] Teramo A Gattazzo C Passeri F Lico A Tasca G Cabrelle A. Intrinsic and Extrinsic Mechanisms Contribute to Maintain the JAK/STAT Pathway Aberrantly Activated in T-Type Large Granular Lymphocyte Leukemia. Blood (2013) 121(19):3843–54, S1. doi: 10.1182/blood-2012-07-441378 23515927

[31] Barilà G Teramo A Calabretto G Ercolin C Boscaro E Trimarco V. Dominant Cytotoxic NK Cell Subset Within CLPD-NK Patients Identifies a More Aggressive NK Cell Proliferation. Blood Cancer J (2018) 8(6):51. doi: 10.1038/s41408-018-0088-1 29891951 PMC6002482

[32] Moignet A Lamy T. Latest Advances in the Diagnosis and Treatment of Large Granular Lymphocytic Leukemia. Am Soc Clin Oncol Educ Book Am Soc Clin Oncol Annu Meet (2018) 38:616–25. doi: 10.1200/EDBK_200689 30231346

[33] Osuji N Matutes E Tjonnfjord G Grech H Del Giudice I Wotherspoon A. T-Cell Large Granular Lymphocyte Leukemia: A Report on the Treatment of 29 Patients and a Review of the Literature. Cancer (2006) 107(3):570–8. doi: 10.1002/cncr.22032 16795070

[34] Go RS Lust JA Phyliky RL. Aplastic Anemia and Pure Red Cell Aplasia Associated With Large Granular Lymphocyte Leukemia. Semin Hematol (2003) 40(3):196–200. doi: 10.1016/s0037-1963(03)00140-9 12876668

[35] Dumitriu B Ito S Feng X Stephens N Yunce M Kajigaya S. Alemtuzumab in T-Cell Large Granular Lymphocytic Leukaemia: Interim Results From a Single-Arm, Open-Label, Phase 2 Study. Lancet Haematol (2016) 3(1):e22–9. doi: 10.1016/S2352-3026(15)00227-6 PMC4721315 26765645

[36] Fortune AF Kelly K Sargent J O’Brien D Quinn F Chadwick N. Large Granular Lymphocyte Leukemia: Natural History and Response to Treatment. Leuk Lymphoma (2010) 51(5):839–45. doi: 10.3109/10428191003706947 20367569

[37] Edelman MJ O’Donnell RT Meadows I. Treatment of Refractory Large Granular Lymphocytic Leukemia With 2-Chlorodeoxyadenosine. Am J Hematol (1997) 54(4):329–31. doi: 10.1002/(sici)1096-8652(199704)54:4<329::aid-ajh13>3.0.co;2-6 9092691

[38] Sternberg A Eagleton H Pillai N Leyden K Turner S Pearson D. Neutropenia and Anaemia Associated With T-Cell Large Granular Lymphocyte Leukaemia Responds to Fludarabine With Minimal Toxicity. Br J Haematol (2003) 120(4):699–701. doi: 10.1046/j.1365-2141.2003.04148.x 12588360

[39] Tsirigotis P Venetis E Kapsimali V Rontogianni D Varvitsioti E Pappa V. 2-Deoxycoformycin in the Treatment of T-Large Granular Lymphocyte Leukemia. Leuk Res (2003) 27(9):865–7. doi: 10.1016/s0145-2126(03)00019-5 12804646

[40] Marchand T Lamy T Finel H Arcese W Choquet S Finke J. Hematopoietic Stem Cell Transplantation for T-Cell Large Granular Lymphocyte Leukemia: A Retrospective Study of the European Society for Blood and Marrow Transplantation. Leukemia (2016) 30(5):1201–4. doi: 10.1038/leu.2015.256 26460210

[41] Bilori B Thota S Clemente MJ Patel B Jerez A Afable Ii M. Tofacitinib as a Novel Salvage Therapy for Refractory T-Cell Large Granular Lymphocytic Leukemia. Leukemia (2015) 29(12):2427–9. doi: 10.1038/leu.2015.280 26449659

[42] Wang TT Yang J Zhang Y Zhang M Dubois S Conlon KC. IL-2 and IL-15 Blockade by BNZ-1, an Inhibitor of Selective γ-Chain Cytokines, Decreases Leukemic T-Cell Viability. Leukemia (2019) 33(5):1243–55. doi: 10.1038/s41375-018-0290-y PMC6478569 30353031

[43] Poh C Arora M Ghuman S Tuscano J. Belinostat in Relapsed/Refractory T-Cell Large Granular Lymphocyte Leukemia. Acta Haematol (2021) 144(1):95–9. doi: 10.1159/000506918 32348994

[44] Lobbes H Dervout C Toussirot E Felten R Sibilia J Wendling D. Rituximab for Rheumatoid Arthritis-Associated Large Granular Lymphocytic Leukemia, a Retrospective Case Series. Semin Arthritis Rheumatol (2020) 50(5):1109–13. doi: 10.1016/j.semarthrit.2020.05.020 32920324

[45] Benjamini O Jain P Konoplev SN Yin CC Abruzzo L Wotherspoon AC. CD4(-)/CD8(-) Variant of T-Cell Large Granular Lymphocytic Leukemia or Hepatosplenic T-Cell Lymphoma: A Clinicopathologic Dilemma. Clin Lymphoma Myeloma Leuk (2013) 13(5):610–3. doi: 10.1016/j.clml.2013.04.010 PMC4167023 23800602

[46] Ahmad E Kingma DW Jaffe ES Schrager JA Janik J Wilson W. Flow Cytometric Immunophenotypic Profiles of Mature Gamma Delta T-Cell Malignancies Involving Peripheral Blood and Bone Marrow. Cytometry B Clin Cytom (2005) 67B(1):6–12. doi: 10.1002/cyto.b.20063 15973700

[47] Chen YH Chadburn A Evens AM Winter JN Gordon LI Chenn A. Clinical, Morphologic, Immunophenotypic, and Molecular Cytogenetic Assessment of CD4–/CD8– γδ T-Cell Large Granular Lymphocytic Leukemia. Am J Clin Pathol (2011) 136(2):289–99. doi: 10.1309/AJCPTFFQ18JMYKDF 21757603

[48] Sanikommu SR Clemente MJ Chomczynski P Afable MG Jerez A Thota S. Clinical Features and Treatment Outcomes in Large Granular Lymphocytic Leukemia (LGLL). Leuk Lymphoma (2018) 59(2):416–22. doi: 10.1080/10428194.2017.1339880 PMC8694069 28633612

[49] Qiu ZY Qin R Tian GY Wang Y Zhang YQ. Pathophysiologic Mechanisms And Management Of Large Granular Lymphocytic Leukemia Associated Pure Red Cell Aplasia. OncoTargets Ther (2019) 12:8229–40. doi: 10.2147/OTT.S222378 PMC6781944 31632073

